# Serum Concentrations of Soluble Flt-1 Are Decreased among Women with a Viable Fetus and No Symptoms of Miscarriage Destined for Pregnancy Loss

**DOI:** 10.1371/journal.pone.0032509

**Published:** 2012-02-28

**Authors:** Tu'uhevaha J. Kaitu'u-Lino, Clare L. Whitehead, Gene-Lyn Ngian, Michael Permezel, Stephen Tong

**Affiliations:** 1 Translational Obstetrics Group, Mercy Hospital for Women, University of Melbourne, Heidelberg, Victoria, Australia; 2 Department of Obstetrics and Gynaecology, Mercy Hospital for Women, University of Melbourne, Heidelberg, Victoria, Australia; Otto-von-Guericke University Magdeburg, Germany

## Abstract

Miscarriage is the most common complication of pregnancy. Pre-clinical miscarriage has an estimated incidence of 30%, whilst clinical miscarriage has an incidence of 12-15%. Two thirds of pregnancies lost to miscarriage are believed to be attributable to defective placentation, thus a number of studies have sought to identify markers of defective placentation that could be used as clinical biomarkers of miscarriage. Decreased soluble FMS-like tyrosine kinase-1 (sFlt1), placental growth factor (PlGF), and soluble endoglin (sEng) in the maternal circulation during the first trimester have recently been proposed as potential markers of pregnancy loss. However, in these studies clinical samples were only obtained once women had presented with symptoms of miscarriage. In this study we prospectively screened serum samples collected from asymptomatic women with a viable fetus. We assessed maternal serum levels of sFlt1, PlGF and sEng across the first trimester of normal pregnancy and compared levels between women who continued to a live birth, to those who subsequently miscarried. Both sFlt1 and PlGF significantly (p≤0.05) increased across gestation in normal pregnancy with serum levels rising from 0.65±0.12 ng/ml at 6 weeks to 1.85±0.24 ng/ml at 12 weeks for sFlt1, and 57.2±19.2 pg/ml to 106±22.7 pg/ml for PlGF. sEng remained unchanged throughout the the first trimester. Importantly we detected a significant (35%, p≤0.05) decrease in sFlt1 levels between our control and miscarriage cohort, however there was significant overlap between cases and controls, suggesting serum sFlt1 is unlikely to be useful as a clinical biomarker in asymptomatic women. Nevertheless, our data suggests a dysregulation of angiogenic factors may be involved in the pathophysiology of miscarriage.

## Introduction

It is well established that early embryo development in the human occurs in a hypoxic environment, and that an increase in placental oxygen levels is observed at the end of the first trimester once maternal blood flow in to the intervillous space is established [1]. Thus throughout the first trimester of normal pregnancies, there is a delicately balanced and tightly controlled state that ensures the developing placenta establishes correctly to allow adequate support and nutrition for the fetus throughout the pregnancy [2].

In the last decade it has been hypothesized that during early development the placenta limits oxygen supply to the developing fetus as a mechanism to protect it against the damaging effects of oxygen free radicals [3]. This protective mechanism is essential to normal development as during this time placental and embryonic cells undergo significant cell division exposing their DNA to potentially deleterious effects of oxidative stress [4].

Pre-clinical pregnancy loss in which conception occurs but ends before delayed menses has an estimated incidence of about 30% [5], whilst clinical pregnancy loss, or clinical miscarriage has an estimated incidence of 12–15% [6], [7]. This means that miscarriage is the most common complication of pregnancy. Two-thirds of pregnancies lost to miscarriage are believed to be attributable to defective placentation associated with an absence of physiological change in maternal spiral arteries and a premature onset of maternal circulation throughout the placenta [2]. This not only causes mechanical damage to the villous tissue but also results in widespread and indirect oxygen mediated trophoblastic damage [2], [8] and eventual expulsion of the conceptus and placenta.

Decreased placental growth factor (PlGF), soluble FMS-like tyrosine kinase -1 (sFlt1) and soluble endoglin (sEng) in the maternal circulation during the first trimester have recently been proposed as potential markers of early pregnancy loss [9], [10], [11], [12]. There is some biological plausibility given sFlt1 and possibly sEng are upregulated by placental hypoxia. sFlt1 is upregulated by cytotrophoblasts under reduced oxygen whilst PlGF is the most abundantly regulated angiogenic factor in uncomplicated first trimester decidua [9].

Interestingly, it was recently reported both sFlt1 and PlGF are significantly decreased in the maternal serum of miscarriage patients compared to asymptomatic controls [11], [12]. Furthermore, in a cohort with threatened miscarriage (vaginal bleeding during pregnancy), those with depressed sFlt1, PlGF and sEng were all significantly more likely to undergo complete miscarriage [11]. However, in all of these studies clinical samples were only obtained once women had presented with symptoms of miscarriage (abdominal pain and bleeding). Therefore, the pathophysiological process of miscarriage in these studies is likely to be already advanced. Potentially, a more useful biomarker would be one that predicts miscarriage among women who are still asymptomatic with a viable fetus. It may be that such a biomarker could flag cases at a higher risk of miscarriage but are at an early stage of the process where potential therapeutic interventions could be successfully administered.

In 2004, we initiated the Mercy Early Pregnancy Study (MEP), a prospective collection of serum samples from asymptomatic women in the first trimester. The primary aim of this study was to identify predictive biomarkers of miscarriage and to validate those previously proposed.

In this study, we used samples selected from the MEP study to examine whether the angiogenic factors sFlt1, PlGF and sEng serum levels are altered in asymptomatic women with viable pregnancies who are destined to miscarry.

## Materials and Methods

### Ethics statement

This study was approved by the Mercy Hospital for Women Ethics committee. All women gave informed written consent to participate in the study.

### Sample collection

Blood samples were collected during The Mercy Early Pregnancy Study, a prospective study designed to identify predictive serum biomarkers of miscarriage among asymptomatic women attending their first prenatal clinic visit between 6 and 12 weeks of gestation [13]. On the day of blood sampling, fetal cardiac activity was confirmed by ultrasound and women had no signs or symptoms of early pregnancy loss. Miscarriage was defined as spontaneous loss of pregnancy at less than 20 weeks gestation.

### Study groups

From the 1044 samples collected during the Mercy Early Pregnancy Study, we selected a case control cohort of 181 samples consisting of 159 controls (women who went on to deliver a live baby at term) and 22 miscarriage samples. Baseline characteristics of all women were recorded including maternal age, gravidity, and parity (Table 1). Gestational age for the miscarriage cohort was calculated from menstrual dates and either menstrual dates or by first trimester ultrasound for controls. Approximately 8 mL of venous blood was collected into vacuette serum clot separator tubes (Greiner Bio-One, Kremsmünster, Austria) without additives. The tubes were centrifuged at 3000 g for 10 minutes, and the serum was collected and stored at −80°C until analysis. All samples were promptly processed and frozen within hours of sample collection.

**Table 1:** Characteristics of study participants.

	Controls (n = 159)	Miscarriage (n = 22)
**Maternal age (years)**	30.0 (21–44)	34.5 (20–43)
**Gravidity[%(n)]**		
**1**	33 (52)	27 (6)
**2**	26 (42)	27 (6)
**≥3**	41 (65)	45 (10)
**Parity [%(n)]**		
**0**	53 (85)	59 (13)
**1**	32 (51)	23 (5)
**≥2**	14 (23)	18 (4)
**Gestation at sampling (wks+days)**	8+4 (6+0−12+6)	7+5 (6+2−10+1)**
**Gestation at 1^st^ symptoms (wks+days)**	n/a	11+0 (7+0−12+3)
**Gestation when the miscarriage was confirmed by ultrasound (wks+days)**	n/a	11+0 (7+0−17+0)
**Gestation at delivery (wks+days)**	39+3 (32+0−42+0)	n/a
**Birth weight (g)**	3493 (1785–4680)	n/a

Data provided as the median, with range given in brackets. p≤0.01 = **.

Samples were divided in to gestational age at the time of blood collection. For control samples, n = 22 at 6 weeks, n = 29 at 7 weeks, n = 33 at 8 weeks, n = 22 at 9 weeks, n = 26 at 10 weeks, n = 21 at 11 weeks, n = 6 at 12 weeks. The gestational age of miscarriage samples were 6–10 weeks with n = 5 at 6 weeks, n = 8 at 7 weeks, n = 6 at 8 weeks, n = 2 at 9 weeks and n = 1 at 10 weeks.

### Immunoassays

Samples were assayed for Soluble Flt-1 (sFlt1), Placental growth factor (PlGF) and soluble endoglin (sEng) using commercially available duoset ELISAs from R&D systems (MN, USA). Proteins were measured in serum samples according to manufacturer's instructions. The minimum detection limit for the respective assays were sFlt1: 39 pg/ml, PlGF: 15.6 pg/ml, sEng: 125 pg/ml.

### Statistical Analysis

For baseline patient characteristics, Mann-Whitney or chi-squared test was used to compare between control and miscarriage groups.

For assessment of differences in protein concentrations across gestation, one-way ANOVAs were performed, with Dunn's post-hoc test indicating differences between gestations. To assess differences between control and miscarriage groups, data was initially corrected for changes across gestation by conversion to MoMs. Mann-Whitney test was then used. P values were considered significant at p<0.05.

## Results

### Patient characteristics

The median maternal age for control women was 30 years, with a range of 21–44. This was not significantly different to the miscarriage cohort (median of 34.5 years, range 20–43, p = 0.46). There was also no significant difference between gravidity and parity between groups with 33%, 27% and 41% of controls having a gravidity of 1, 2 or 3 respectively compared to 27%, 27% and 45% for the miscarriage cohort (p = 0.87, chi-squared test). For parity in the control group 53% were nulliparous, 32% were primiparous, and 14% had 2 or more live births compared to 59%, 23% and 18% for the miscarriage cohort. The median gestation at sampling for miscarriage patients was significantly lower than control. Miscarriage samples did not include any 11 or 12 week samples, and thus for multiples of the median comparisons (MoM) only 6–10 week control samples were included.

For the miscarriage cohort, 12/22 patients attended the hospital with symptoms of miscarriage, whilst 10/22 were asymptomatic prior to diagnosis. n = 18 of the miscarriage patients were diagnosed in the first trimester, n = 4 in the 2^nd^ trimester. The median gestation when the samples were taken was over three weeks from the date when the women suffered their 1^st^ symptoms of miscarriage and/or were diagnosed with a miscarriage (table 1).

### sFlt1 and PlGF increase across early pregnancy in control samples

**Figure 1:**
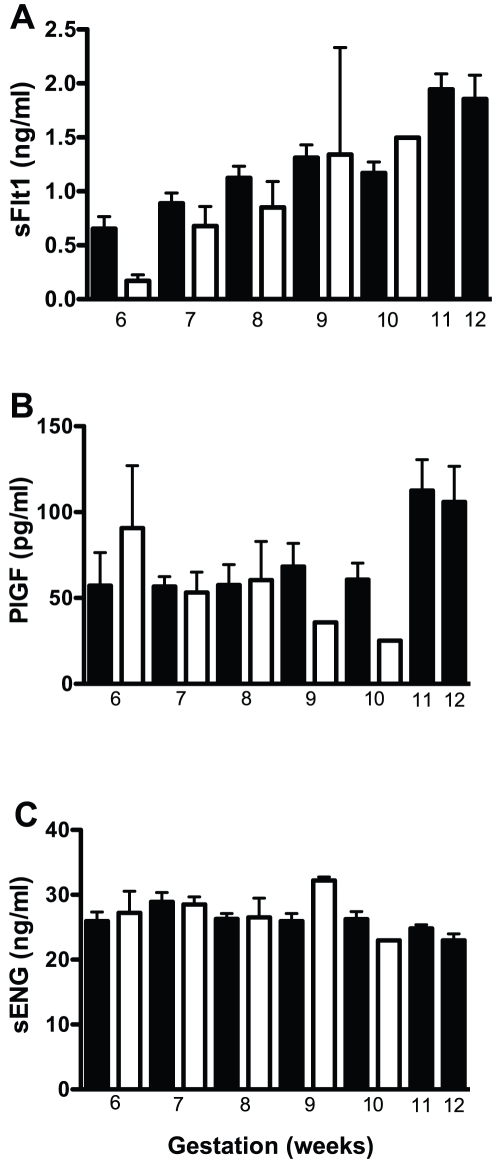
Analysis of sFlt1 (A), PlGF (B) and sEng (C) in maternal serum from normal pregnancies (black bars) and pregnancies that went onto miscarry (white bars) throughout the first trimester. In normal pregnancies, a non-parametric ANOVA revealed that sFlt1 and PlGF significantly (p≤0.05) increased throughout the first trimester whilst sEng did not change. No differences were detected between control and miscarriage groups at each gestation. Data expressed as mean+ S.E.M.

sFlt1 serum concentrations rose significantly (p≤0.05) across gestation during early pregnancy with mean serum concentrations (± SEM) being 0.65±0.12 ng/ml at 6 weeks, 0.89±0.1 ng/ml at 7 weeks, 1.12±0.11 ng/ml at 8 weeks, 1.31±0.12 ng/ml at 9 weeks, 1.17±0.1 ng/ml at 10 weeks, 1.94±0.15 ng/ml at 11 weeks and 1.85±0.24 ng/ml at 12 weeks (Figure 1A).

PlGF serum concentrations significantly rose (p≤0.05) across gestation during early pregnancy with mean serum concentrations (± SEM) being 57.2±19.2 pg/ml at 6 weeks, 50.4±5.8 pg/ml at 7 weeks, 57.5±12 pg/ml at 8 weeks, 68.3±13.5 pg/ml at 9 weeks, 60.7±9.6 pg/ml at 10 weeks, 112.6±17.9 pg/ml at 11 weeks and 106±22.7 pg/ml at 12 weeks (Figure 1B).

sEng serum concentrations remained unchanged throughout early pregnancy with mean serum concentrations (±SEM) being 26±1.4 ng/ml at 6 weeks, 28.9±1.4 ng/ml at 7 weeks, 26.3±0.8 ng/ml at 8 weeks, 26±1.2 ng/ml at 9 weeks, 26.3±1.2 ng/ml at 10 weeks, 24.8±0.6 ng/ml at 11 weeks and 23±1.1 ng/ml at 12 weeks (Figure 1C).

Given sFlt1 is capable of antagonizing PlGF, we carried out a correlation between sFlt1 and PlGF to further assess the potential relationship between the two throughout the first trimester (data not shown), however identified no relationship between the two factors in normal pregnancies when assessing either the raw values (r^2^ = 0.06) or the MoMs (r^2^ = 0.02).

### sFlt1, but not PlGF or sEng is decreased preceding symptoms of miscarriage

**Figure 2:**
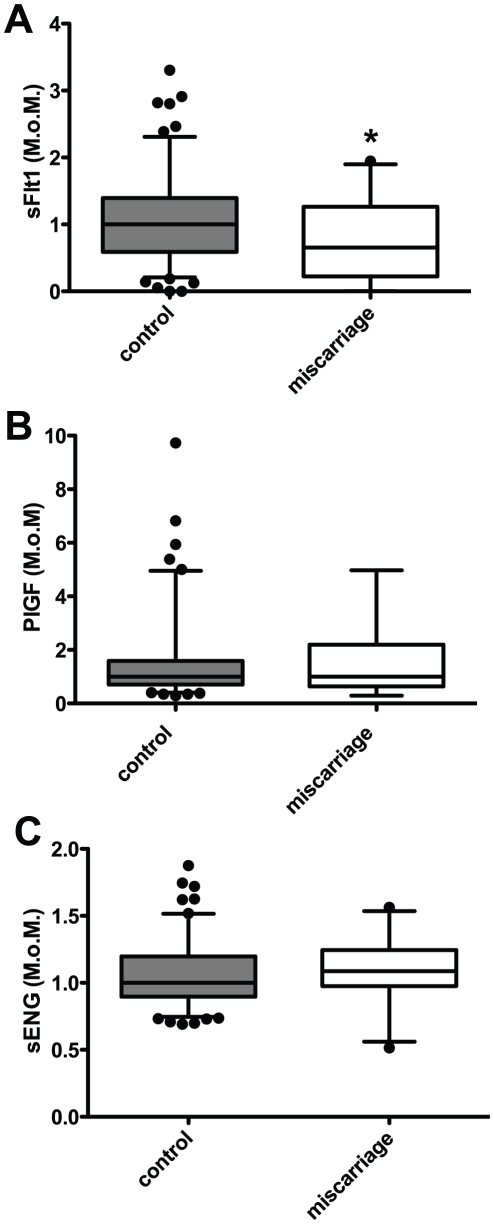
Analysis of MoM for serum concentrations of sFlt1 (A), PlGF (B), and sEng (C) between control and miscarriage cohorts. sFlt1 was signifcantly decreased in the miscarraige cohort compared to control, whilst no change in PlGF or sEng was detected. Data is displayed as a box and whisker's plot with median and 5–95^th^ percentiles shown. p≤0.05 = *.

There was a significant decrease (35%, p<0.05) in circulating sFlt1 (Figure 2A) in serum among the miscarriage cohort from women who went on to miscarry compared to women who delivered a live baby at term (control MoM = 1.0 (range 0–3.3), vs 0.65 (range 0–1.94). In contrast, no change was observed in circulating PlGF or sEng between the two groups (Figure 2B, 2C). No significant differences were detected between control and miscarriage groups at each gestational age from 6 to 10 weeks gestation for sFlt1, PlGF or sEng (Figure 1).

The Receiver Operator Curve (ROC) Area under the curve (AUC) for sFlt1 was 0.66 (p = 0.03). Therefore, despite a statistically significant difference in sFlt1 between the miscarriage cohort and controls, sFLt1 has a poor sensitivity and specificity for predicting miscarriage in asymptomatic women (data not shown).

## Discussion

In this study, we show that early pregnancy maternal sFlt-1 but not PlGF or sEng levels are significantly lower in asymptomatic women who go on to miscarry compared to women who progress to a live birth. However, there is significant overlap between cases and controls, suggesting serum sFlt1 is unlikely to be useful as a clinical biomarker. Nevertheless, the depression of sFlt1 in association with miscarriage provides interesting biological insights.

Although sFlt1 has recently been proposed as a biomarker of miscarriage [11], [12], our study is novel given it was a large prospective study recruiting an asymptomatic cohort in the mid-first trimester with confirmation of fetal viability at the time of sample collection. We believe this is a clinically useful stage of pregnancy where prediction of miscarriage may be most useful. Most previous reports investigating the ability of blood biomarkers to predict miscarriage have been performed among cohorts already experiencing symptoms of miscarriage (bleeding and abdominal pain) [11], [12], [14]. However, development of a clinical biomarker in this setting is arguably of limited clinical utility given by this stage, the most appropriate investigation is serial ultrasounds. Furthermore, the biological information that could be inferred from such studies are limited by the fact the pathophysiology of miscarriage is already advanced (bleeding into subchorionic spaces leading to vaginal bleeding). In contrast we believe our samples reflect the endocrinology of miscarriage early in its pathogenesis when the fetus is still viable and the women is yet to experience symptoms of miscarriage.

sFlt1 and PlGF have often been studied in the maternal serum for their predictive value in diseases of pregnancy such as preeclampsia, intrauterine growth restriction and small for gestational age infants [10], [15], [16]. In these conditions, sFlt1 increases in association with pathology whilst PlGF decreases. This is the first study that has documented the week-by-week changes in serum PlGF, sFlt1 and sEng across the mid first trimester (6–11 weeks).

Although generally assessed in later pregnancy, a rise in PlGF throughout pregnancy in the maternal serum of normal pregnancies is well established [16], [17]. One study in early pregnancy suggested that rises in PlGF are associated with increased placental perfusion and placental mass [18] which would account for the high levels we observed at 11 and 12 weeks compared to 6–10 weeks gestation. Moreover, PlGF is predominantly expressed in the syncytiotrophoblast, which has direct contact with the maternal circulation [19] thus allowing release of high levels of PlGF into the maternal circulation as the placental mass increases. Interestingly it has also been shown that higher levels of PlGF in early pregnancy are associated with a decreased risk of disease in later pregnancy, suggesting that low circulating PlGF may be indicative of defective placentation [15].

Although high levels of sFlt1 are associated with pre-eclampsia in later pregnancy [20], in normal first trimester pregnancies high levels of sFlt1 are believed to be physiological and a result of excessive placental production under hypoxic conditions [21]. sFlt1 is an anti-angiogenic factor that antagonizes both VEGF and PlGF. Free VEGF is undetectable in maternal serum, presumably due to it being bound by sFlt1, whilst high circulating sFlt1 has been associated with lower circulating PlGF [16]. While of uncertain significance, it is intriguing we observed a sudden increase in serum sFlt1 and PlGF concentrations at 11 and 12 weeks correlates with a period where trophoblast plugs dislodge and there is sudden perfusion of the intervillous space. This sudden perfusion is thought to render the placenta less hypoxic than in earlier pregnancy.

We were able to detect the presence of sEng but observed no change in levels across 6–12 weeks gestation, a time when there is increasing placental mass. This raises the possibility that in early pregnancy serum sEng may be from an extra-placental source such as the maternal endothelium.

Two studies [11], [22] have recently flagged sFlt1 and PlGF as potential biomarkers of early pregnancy loss using samples collected from women presenting with symptoms of miscarriage such as abdominal pain or vaginal bleeding. In agreement with these, our analysis of MoMs clearly indicated a decline in sFlt1 in samples from pregnancies that went on to miscarry compared to those who went on to produce live offspring. This is particularly interesting as it is suggestive of defective placentation before the onset of symptoms of miscarriage. Given the delicate balance that promotes placentation under hypoxic conditions in normal early pregnancies, including successful angiogenesis and regulation of pro- and anti- angiogenic factors such as VEGF and sFlt1, our findings suggest an imbalance of pro and anti-angiogenic factors may be an early step in the pathophysiological process of miscarriage which, in turn, may be precipitated by differences in oxygen tension. However, in contrast to previous studies that have examined a symptomatic cohort [11], [12], our study suggests sFlt1 is unlikely to be a clinical useful predictor of miscarriage among asymptomatic women in the first trimester.
